# Top-cited articles in cerebrospinal fluid leak (rhinorrhea and otorrhea) (1945–2018)

**DOI:** 10.1016/j.bjorl.2019.12.002

**Published:** 2020-01-14

**Authors:** Irene Monjas-Cánovas, Isabel Belinchón-Romero, Juan-Ramón Gras-Albert, Gregorio González-Alcaide, José Manuel Ramos-Rincón

**Affiliations:** a General University Hospital of Alicante, Department of Otorhinolaryngology, Alicante, Spain; b Miguel Hernandez University of Elche, Department of Clinical Medicine, Alicante, Spain; c Independent Researcher, Alicante, Spain; d University of Valencia, Department of History of Science and Documentation, Valencia, Spain

**Keywords:** Bibliometric, publications, Cerebrospinal fluid leak, Cerebrospinal fluid rhinorrhea, Cerebrospinal fluid otorrhea

## Abstract

**Introduction:**

As scientific knowledge has grown in biomedicine, it has also become necessary to develop tools to manage and understand the body of evidence. In that sense, bibliometrics has become a consolidated discipline for analyzing scientific activity, enabling the characterization of a particular field or area of knowledge by means of the quantification of the bibliographic characteristics of scientific publications.

**Objective:**

The objective of this study was to determine the most frequently cited articles in the field of cerebrospinal fluid rhinorrhea and otorrhea.

**Methods:**

The searches took place on the Clarivate Analytics Web of Science platform, which includes the MEDLINE database. The study period was limited to 1945–2018.

**Results:**

The 101 most cited articles in the field of cerebrospinal fluid leak were published in 36 journals, and the most important specialties contributing to the literature were neurosurgery and otorhinolaryngology. Of the 101 top-cited articles, 70% were published from 1990 to 2018, with two distinct periods of high scientific productivity: 1990–1999 and 2000–2009. In the first period, the main topic of research interest was endoscopic sinus surgery for cerebrospinal fluid fistulas, whereas from 2000 to 2009, documents focused more on surgical aspects of extended skull base approaches. The articles received 73–767 citations. The top article over the whole study period was “A novel reconstructive technique after endoscopic expanded endonasal approaches: vascular pedicle nasoseptal flap” by Hadad et al., which was published 2006 in Laryngoscope. Its publication represented an inflection point in the literature on cerebrospinal fluid leak and endoscopic skull base surgery, and it gave rise to numerous other research publications.

**Conclusion:**

Different surgical innovations in the field of cerebrospinal fluid leak sparked two different periods of intense scientific activity. Otorhinolaryngology and neurosurgery were the dominant specialties. The most frequent topic studied was endoscopic surgery; others included clinical and diagnostic features, neurinoma surgery, and cerebrospinal fluid leak related to temporal bone fractures.

## Introduction

Cerebrospinal fluid (CSF) leak describes the discharge of CSF from the intracranial cavity through an osseous defect within the skull base. The underlying dura mater and adherent pia-arachnoid mater are disrupted, resulting in a communication between the intracranial cavity, the subarachnoid space and either the nasal or middle ear cavity. The condition was first described as a pathologic entity in 1899 by Clair Thompson.^1^ While CSF leaks may occur spontaneously, common etiologies of CSF rhinorrhea include trauma, neoplasms, and prior surgery, while CSF otorrhea is usually associated with craniocerebral trauma (e.g. skull fracture involving the temporal bone), neurosurgical procedures, or other conditions.^2^ Patients with CSF leak can present with a variety of symptoms such as clear nasal discharge and headache or complications such as pneumocephalus, meningitis or brain abscess.

As scientific knowledge has grown in biomedicine, it has also become necessary to develop tools to manage and understand the body of evidence. In that sense, bibliometrics has become a consolidated discipline for analyzing scientific activity, enabling the characterization of a particular field or area of knowledge by means of the quantification of the bibliographic characteristics of scientific publications.^2^, ^3^ Nowadays, identifying citation classics and top-cited papers is one of the key methodologies used to systematically evaluate research performance. This information can help optimize the allocation of resources, reorient research support, rationalize research organizations, restrict research in particular fields, and augment research productivity.^3^ Across different fields, the scope of scientific literature has broadened to achieve a more multidisciplinary vision. This expanded focus justifies the review of the most important papers to help guide future research and practice. In that sense, several medical specialties have ranked articles within their fields by citation frequency.^4^, ^5^, ^6^, ^7^

In otorhinolaryngology, several factors have contributed to a growth in research output: the academic development of the field and training programs; significant advances in information and communication technology, which allow studies and experiments to be rapidly performed, written, reviewed, published, and cited; the increase in cooperative practices and multidisciplinary research approaches; the growing need to publish in order to secure academic promotions and research funding; and the existence of a larger critical mass and evidence base, driving further research advances in the field.^8^, ^9^ Research on the etiology, diagnosis and treatment for CSF leak has also increased over the last few decades, but while several studies have analyzed the top cited documents in otorhinolaryngology,^4^, ^10^, ^11^, ^12^ none have specifically focused on CSF leak.

The present study identifies and analyzes the characteristics of the 100 most cited articles on CSF rhinorrhea and otorrhea. This information could help researchers and professionals understand the research areas that are generating the most impact on the field, the countries that are making the largest contribution, and the main journals used to disseminate advances.

## Methods

We opted to identify documents about CSF leak by means of the Medical Subject Headings (MeSH) thesaurus, a detailed instrument for controlled terminology. The MEDLINE database included the terms “Cerebrospinal fluid rhinorrhea” and “Cerebrospinal fluid otorrhea” in 1966 to define respective CSF discharges through the nose or through the external auditory meatus/eustachian tube into the nasopharynx. However, it was not until 2005 that the database included a composite descriptor for “Cerebrospinal fluid leak” to define any discharge of CSF through a hole in the skull bone.

The study period was limited to 1945–2018. The searches took place on the Clarivate Analytics Web of Science (WoS) platform, which includes MEDLINE database, on January 21, 2019. The WoS Core Collection provides information on the number of times a particular article has been cited by other articles. This strategy yielded a total of 4155 documents from the MEDLINE database. After excluding 25 editorials and 1021 documents that were not indexed in the WoS Core Collection, we had a total of 3109 articles, from which we aimed to identify the 100 most cited papers. In fact, we included 101 articles because the papers ranked 100 and 101 received the same numbers of citations. Data collected for each article included the full reference (author’s names, journal title and publication year), impact factor (in the Journal Citation Report [JCR] 2017), WoS category of the journal, countries of authors, and type of document (article, review, case report, meta-analysis, trial). Citation density was determined by dividing the number of citations received by the position of the journal in the JCR category.

## Results

Table 1 lists the full references and citation data for the 101 top-cited articles in the literature on CSF leak. Laryngoscope was the most prolific journal, publishing 20 articles, followed by the *Journal of* Neurosurgery (n = 17), Neurosurgery (n = 12), Annals of Otology, Rhinology & Laryngology (n = 6), and Otolaryngology-Head and Neck Surgery (n = 6). These 5 journals covered 60.4% of the highest-impact documents (Table 2).

**Table 1:** Distribution of the top 101 cited-papers on Cerebrospinal Fluid Leak group with country. citations and citation density.

Rank	Publication	Country of origin, 1st author	Countries of origin, rest of authors	Citations (n)	Citation density
1	Hadad G, Bassagasteguy L, Carrau RL, Mataza JC, Kassam A, Snyderman CH, Mintz A. A novel reconstructive technique after endoscopic expanded endonasal approaches: vascular pedicle nasoseptal flap. Laryngoscope. 2006;116(10):1882-6.	USA	Argentina	767	69.7
			USA		
2	Hegazy HM, Carrau RL, Snyderman CH, Kassam A, Zweig J. Transnasal endoscopic repair of cerebrospinal fluid rhinorrhea: a meta-analysis. Laryngoscope. 2000;110(7):1166-72.	USA	USA	272	16.0
3	Kassam AB, Prevedello DM, Carrau RL, Snyderman CH, Thomas A, Gardner P, Zanation A, Duz B, Stefko ST, Byers K, Horowitz MB. Endoscopic endonasal skull base surgery: analysis of complications in the authors' initial 800 patients. J Neurosurg. 2011;114(6):1544-68.	USA	USA	268	44.7
			Turkey		
4	Ommaya AK, Di Chiro G, Baldwin M, Pennybacker JB. Non-traumatic cerebrospinal fluid rhinorrhea. J Neurol Neurosurg Psychiatry. 1968;31(3):214-25.	USA	USA	252	5.1
5	Black PM, Zervas NT, Candia GL. Incidence and management of complications of trans-phenoidal operation for pituitary adenomas. Neurosurgery.1987;20(6):920-4.	USA	USA	248	8.3
6	Gormley WB, Sekhar LN, Wright DC, Kamerer D, Schessel D. Acoustic neuromas: results of current surgical management. Neurosurgery. 1997;41(1):50-8; discussion 58–60.	USA	USA	222	11.1
7	Mattox DE, Kennedy DW. Endoscopic management of cerebrospinal fluid leaks and cephaloceles. Laryngoscope. 1990;100(8):857-62.	USA	USA	201	7.4
8	May M, Levine HL, Mester SJ, Schaitkin B. Complications of endoscopic sinus surgery: analysis of 2108 patients--incidence and prevention. Laryngoscope. 1994;104(9):1080-3.	USA	USA	200	8.7
9	Lanza DC, O'Brien DA, Kennedy DW. Endoscopic repair of cerebrospinal fluid fistulae and encephaloceles. Laryngoscope. 1996;106(9 Pt 1):1119-25.	USA	USA	200	9.5
10	Frank G, Pasquini E, Doglietto F, Mazzatenta D, Sciarretta V, Farneti G, Calbucci F. The endoscopic extended trans-phenoidal approach for craniopharyngiomas. Neurosurgery. 2006 Jul;59(1 Suppl 1):ONS75-83; discussion ONS75-83.	Italy	Italy	175	15.9
11	Brodie HA, Thompson TC. Management of complications from 820 temporal bone fractures. Am J Otol. 1997;18(2):188-97.	USA	USA	166	8.3
12	Esposito F, Dusick JR, Fatemi N, Kelly DF. Graded repair of cranial base defects and cerebrospinal fluid leaks in transsphenoidal surgery. Neurosurgery. 2007;60(4 Suppl 2):295-303; discussion 303-4.	USA	Italy	150	15.0
13	Hosobuchi Y. Direct surgical treatment of giant intracranial aneurysms. J Neurosurg. 1979;51(6):743-56.	USA	USA	149	3.9
14	Hubbard JL, McDonald TJ, Pearson BW, Laws ER Jr. Spontaneous cerebrospinal fluid rhinorrhea: evolving concepts in diagnosis and surgical management based on the Mayo Clinic experience from 1970 through 1981. Neurosurgery. 1985;16(3):314-21.	USA	USA	144	4.5
15	Boulware DR, Meya DB, Muzoora C, Rolfes MA, Huppler Hullsiek K, Musubire A, Taseera K, Nabeta HW, Schutz C, Williams DA, Rajasingham R, Rhein J, Thienemann F, Lo MW, Nielsen K, Bergemann TL, Kambugu A, Manabe YC, Janoff EN, Bohjanen PR, Meintjes G; COAT Trial Team. Timing of antiretroviral therapy after diagnosis of cryptococcal meningitis. N Engl J Med. 2014 26;370(26):2487-98.	USA	Uganda	140	46.7
			South Africa		
			USA		
			UK		
16	Lanman TH, Brackmann DE, Hitselberger WE, Subin B. Report of 190 consecutive cases of large acoustic tumors (vestibular schwannoma) removed via the translabyrinthine approach. J Neurosurg. 1999;90(4):617-23.	USA	USA	139	7.7
17	Park JI, Strelzow VV, Friedman WH. Current management of cerebrospinal fluid rhinorrhea. Laryngoscope. 1983;93(10):1294-300.	USA	USA	128	3.8
18	Phelps PD, Reardon W, Pembrey M, Bellman S, Luxom L. X-linked deafness, stapes gushers and a distinctive defect of the inner ear. Neuroradiology. 1991;33(4):326-30.	UK	UK	125	4.8
19	LEWIN W. Cerebrospinal fluid rhinorrhoea in closed head injuries. Br J Surg. 1954;42(171):1-18.	UK	‒	124	2.0
20	Harvey RJ, Parmar P, Sacks R, Zanation AM. Endoscopic skull base reconstruction of large dural defects: a systematic review of published evidence. Laryngoscope. 2012;122(2):452-9.	Australia	USA	122	24.4
21	Freedman HM, Kern EB. Complications of intranasal ethmoidectomy: a review of 1000 consecutive operations. Laryngoscope. 1979;89(3):421-34.	USA	USA	122	3.2
22	Casiano RR, Jassir D. Endoscopic cerebrospinal fluid rhinorrhea repair: is a lumbar drain necessary? Otolaryngol Head Neck Surg. 1999;121(6):745-50.	USA	USA	118	6.6
23	Zweig JL, Carrau RL, Celin SE, Schaitkin BM, Pollice PA, Snyderman CH, Kassam A, Hegazy H. Endoscopic repair of cerebrospinal fluid leaks to the sinonasal tract: predictors of success. Otolaryngol Head Neck Surg. 2000;123(3):195-201.	USA	USA	116	6.8
24	Shapiro SA, Scully T. Closed continuous drainage of cerebrospinal fluid via a lumbar subarachnoid catheter for treatment or prevention of cranial/spinal cerebrospinal fluid fistula. Neurosurgery. 1992;30(2):241-5.	USA	USA	114	4.6
25	Stone JA, Castillo M, Neelon B, Mukherji SK. Evaluation of CSF leaks: high-resolution CT compared with contrast-enhanced CT and radionuclide cisternography. AJNR Am J Neuroradiol. 1999;20(4):706-12.	USA	USA	114	6.3
26	Cannon CR, Jahrsdoerfer RA. Temporal bone fractures. Review of 90 cases. Arch Otolaryngol. 1983;109(5):285-8.	USA	USA	114	3.4
27	Cappabianca P, Cavallo LM, Esposito F, Valente V, De Divitiis E. Sellar repair in endoscopic endonasal transsphenoidal surgery: results of 170 cases. Neurosurgery. 2002;51(6):1365-71; discussion 1371-2.	Italy	Italy	112	7.5
28	Banks CA, Palmer JN, Chiu AG, O'Malley BW Jr, Woodworth BA, Kennedy DW. Endoscopic closure of CSF rhinorrhea: 193 cases over 21 years. Otolaryngol Head Neck Surg. 2009;140(6):826-33.	USA	USA	110	13.8
29	Kassam A, Thomas AJ, Snyderman C, Carrau R, Gardner P, Mintz A, Kanaan H, Horowitz M, Pollack IF. Fully endoscopic expanded endonasal approach treating skull base lesions in pediatric patients. J Neurosurg. 2007;106(2 Suppl):75-86.	USA	USA	108	10.8
30	Narotam PK, van Dellen JR, Bhoola KD. A clinicopathological study of collagen sponge as a dural graft in neurosurgery. J Neurosurg. 1995;82(3):406-12.	South Africa	South Africa	108	4.9
31	Shetty PG, Shroff MM, Fatterpekar GM, Sahani DV, Kirtane MV. A retrospective analysis of spontaneous sphenoid sinus fistula: MR and CT findings. AJNR Am J Neuroradiol. 2000;21(2):337-42.	India	India	108	6.4
32	Shetty PG, Shroff MM, Sahani DV, Kirtane MV. Evaluation of high-resolution CT and MR cisternography in the diagnosis of cerebrospinal fluid fistula. AJNR Am J Neuroradiol. 1998;19(4):633-9.	India	India	107	5.6
33	Jho HD, Ha HG. Endoscopic endonasal skull base surgery: Part 1-The midline anterior fossa skull base. Minim Invasive Neurosurg. 2004;47(1):1-8.	USA	USA	107	8.2
34	Stankiewicz JA. Cerebrospinal fluid fistula and endoscopic sinus surgery. Laryngoscope. 1991;101(3):250-6.	USA	‒	105	4.0
35	Cohen NL, Lewis WS, Ransohoff J. Hearing preservation in cerebellopontine angle tumor surgery: the NYU experience 1974-1991. Am J Otol. 1993;14(5):423-33.	USA	USA	105	4.4
36	Yonekawa Y, Ogata N, Imhof HG, Olivecrona M, Strommer K, Kwak TE, Roth P,Groscurth P. Selective extradural anterior clinoidectomy for supra- and parasellar processes. Technical note. J Neurosurg. 1997;87(4):636-42.	Switzerland	Switzerland	102	5.1
37	Laws ER Jr, Fode NC, Redmond MJ. Transsphenoidal surgery following unsuccessful prior therapy. An assessment of benefits and risks in 158 patients. J Neurosurg. 1985;63(6):823-9.	USA	USA	101	3.2
38	Schlosser RJ, Woodworth BA, Wilensky EM, Grady MS, Bolger WE. Spontaneous cerebrospinal fluid leaks: a variant of benign intracranial hypertension. Ann Otol Rhinol Laryngol. 2006;115(7):495-500.	USA	USA	101	9.2
39	Hoffman RA. Cerebrospinal fluid leak following acoustic neuroma removal. Laryngoscope. 1994;104(1 Pt 1):40-58.	USA	‒	101	4.4
40	Darrouzet V, Martel J, Enée V, Bébéar JP, Guérin J. Vestibular schwannoma surgery outcomes: our multidisciplinary experience in 400 cases over 17 years. Laryngoscope. 2004;114(4):681-8.	France	France	100	7.7
41	Schlosser RJ, Bolger WE. Nasal cerebrospinal fluid leaks: critical review and surgical considerations. Laryngoscope. 2004;114(2):255-65.	USA	USA	99	7.6
42	Berker M, Hazer DB, Yücel T, Gürlek A, Cila A, Aldur M, Onerci M. Complications of endoscopic surgery of the pituitary adenomas: analysis of 570 patients and review of the literature. Pituitary. 2012;15(3):288-300.	Turkey	Turkey	97	19.4
43	Schlosser RJ, Wilensky EM, Grady MS, Bolger WE. Elevated intracranial pressures in spontaneous cerebrospinal fluid leaks. Am J Rhinol. 2003;17(4):191-5.	USA	USA	97	6.9
44	Mortara R, Norrell H. Consequences of a deficient sellar diaphragm. J Neurosurg. 1970;32(5):565-73.	USA	USA	97	2.1
45	Jane JA Jr, Thapar K, Kaptain GJ, Maartens N, Laws ER Jr. Pituitary surgery: transsphenoidal approach. Neurosurgery. 2002;51(2):435-42; discussion 442-4.	USA	Canada	96	6.4
			USA		
46	MacGee EE, Cauthen JC, Brackett CE. Meningitis following acute traumatic cerebrospinal fluid fistula. J Neurosurg. 1970;33(3):312-6.	USA	USA	96	2.0
47	Yasargil MG, Fox JL. The microsurgical approach to acoustic neurinomas. Surg Neurol. 1974;2(6):393-8.	Switzerland	Switzerland	96	2.2
48	Di Chiro G, Ommaya AK, Ashburn WL, Briner WH. Isotope cisternography in the diagnosis and follow-up of cerebrospinal fluid rhinorrhea. J Neurosurg. 1968;28(6):522-9.	USA	USA	95	1.9
49	D'Haens J, Van Rompaey K, Stadnik T, Haentjens P, Poppe K, Velkeniers B. Fully endoscopic transsphenoidal surgery for functioning pituitary adenomas: a retrospective comparison with traditional transsphenoidal microsurgery in the same institution. Surg Neurol. 2009;72(4):336-40.	Belgium	Belgium	95	11.9
50	Woodworth BA, Prince A, Chiu AG, Cohen NA, Schlosser RJ, Bolger WE, Kennedy DW, Palmer JN. Spontaneous CSF leaks: a paradigm for definitive repair and management of intracranial hypertension. Otolaryngol Head Neck Surg. 2008;138(6):715-20.	USA	USA	92	10.2
51	Morales F, Mostacero E, Marta J, Sanchez S. Vascular malformation of the cerebellopontine angle associated with "SUNCT" syndrome. Cephalalgia. 1994;14(4):301-2.	Spain	Spain	92	4.0
52	Gacek RR, Gacek MR, Tart R. Adult spontaneous cerebrospinal fluid otorrhea: diagnosis and management. Am J Otol. 1999;20(6):770-6.	USA	USA	91	5.1
53	Shah RN, Surowitz JB, Patel MR, Huang BY, Snyderman CH, Carrau RL, Kassam AB, Germanwala AV, Zanation AM. Endoscopic pedicled nasoseptal flap reconstruction for pediatric skull base defects. Laryngoscope. 2009;119(6):1067-75.	USA	USA	91	11.4
54	Maira G, Anile C, Albanese A, Cabezas D, Pardi F, Vignati A. The role of transsphenoidal surgery in the treatment of craniopharyngiomas. J Neurosurg. 2004;100(3):445-51.	Italy	Italy	90	6.9
55	Keerl R, Weber RK, Draf W, Wienke A, Schaefer SD. Use of sodium fluorescein solution for detection of cerebrospinal fluid fistulas: an analysis of 420 administrations and reported complications in Europe and the United States. Laryngoscope. 2004;114(2):266-72.	Germany	USA	90	6.9
56	Stankiewicz JA. Complications of endoscopic sinus surgery. Otolaryngol Clin North Am. 1989;22(4):749-58.	USA	‒	90	3.2
57	Dodson EE, Gross CW, Swerdloff JL, Gustafson LM. Transnasal endoscopic repair of cerebrospinal fluid rhinorrhea and skull base defects: a review of twenty-nine cases. Otolaryngol Head Neck Surg. 1994;111(5):600-5.	USA	USA	90	3.9
58	Romeo MJ, Espina V, Lowenthal M, Espina BH, Petricoin EF 3^rd^, Liotta LA. CSF proteome: a protein repository for potential biomarker identification. Expert Rev Proteomics. 2005;2(1):57-70.	USA	USA	89	7.4
59	Glasscock ME 3^rd^. The stapes gusher. Arch Otolaryngol. 1973;98(2):82-91.	USA	‒	89	2.0
60	Carrau RL, Snyderman CH, Kassam AB. The management of cerebrospinal fluid leaks in patients at risk for high-pressure hydrocephalus. Laryngoscope. 2005;115(2):205-12.	USA	USA	88	7.3
61	Brodie HA. Prophylactic antibiotics for posttraumatic cerebrospinal fluid fistulae. A meta-analysis. Arch Otolaryngol Head Neck Surg. 1997;123(7):749-52.	USA	‒	88	4.4
62	Lindstrom DR, Toohill RJ, Loehrl TA, Smith TL. Management of cerebrospinal fluid rhinorrhea: the Medical College of Wisconsin experience. Laryngoscope. 2004;114(6):969-74.	USA	USA	87	6.7
63	Cohen NL, Hammerschlag P, Berg H, Ransohoff J. Acoustic neuroma surgery: an eclectic approach with emphasis on preservation of hearing. The New York University-Bellevue experience. Ann Otol Rhinol Laryngol. 196;95(1 Pt1):21-7.	USA	USA	87	2.8
64	Ray BS, Bergland RM. Cerebrospinal fluid fistula: clinical aspects, techniques of localization, and methods of closure. J Neurosurg. 1969;30(4):399-405.	USA	USA	87	1.8
65	Drayer BP, Wilkins RH, Boehnke M, Horton JA, Rosenbaum AE. Cerebrospinal fluid rhinorrhea demonstrated by metrizamide CT cisternography. AJR Am J Roentgenol. 1977;129(1):149-51.	USA	USA	86	2.2
66	Gacek RR, Leipzig B. Congenital cerebrospinal otorrhea. Ann Otol Rhinol Laryngol. 1979;88(3 Pt 1):358-65.	USA	USA	86	2.3
67	Hardy DG, Macfarlane R, Baguley D, Moffat DA. Surgery for acoustic neurinoma. An analysis of 100 translabyrinthine operations. J Neurosurg. 1989;71(6):799-804.	UK	UK	86	3.1
68	Sekhar LN, Schessel DA, Bucur SD, Raso JL, Wright DC. Partial labyrinthectomy petrous apicectomy approach to neoplastic and vascular lesions of the petroclival area. Neurosurgery. 1999;44(3):537-50; discussion 550-2.	USA	USA	86	4.8
69	Komotar RJ, Starke RM, Raper DM, Anand VK, Schwartz TH. Endoscopic endonasal versus open transcranial resection of anterior midline skull base meningiomas. World Neurosurg. 2012;77(5–6):713-24.	USA	USA	85	17.0
			Australia		
70	Amico JA, Tenicela R, Johnston J, Robinson AG. A time-dependent peak of oxytocin exists in cerebrospinal fluid but not in plasma of humans. J Clin Endocrinol Metab. 1983;57(5):947-51.	USA	USA	84	2.5
71	Mokri B. Spontaneous low pressure, low CSF volume headaches: spontaneous CSF leaks. Headache. 2013;53(7):1034-53.	USA	‒	83	20.8
72	Gacek RR. Arachnoid granulation cerebrospinal fluid otorrhea. Ann Otol Rhinol Laryngol. 1990;99(11):854-62.	USA		83	3.1
73	Kaufman B, Nulsen FE, Weiss MH, Brodkey JS, White RJ, Sykora GF. Acquired spontaneous, nontraumatic normal-pressure cerebrospinal fluid fistulas originating from the middle fossa. Radiology. 1977;122(2):379-87.	USA	USA	82	2.1
74	Simasek M, Blandino DA. Treatment of the common cold. Am Fam Physician. 2007;15;75(4):515-20.	USA	USA	82	8.2
75	Friedman JA, Ebersold MJ, Quast LM. Post-traumatic cerebrospinal fluid leakage. World J Surg. 2001;25(8):1062-6.	USA	USA	82	5.1
76	Wormald PJ, McDonogh M. 'Bath-plug' technique for the endoscopic management of cerebrospinal fluid leaks. J Laryngol Otol. 1997;111(11):1042-6.	China		81	4.1
77	Ramakrishnan VR, Kingdom TT, Nayak JV, Hwang PH, Orlandi RR. Nationwide incidence of major complications in endoscopic sinus surgery. Int Forum Allergy Rhinol. 2012;2(1):34-9.	USA	USA	81	16.2
78	Leech PJ, Paterson A. Conservative and operative management for cerebrospinal-fluid leakage after closed head injury. Lancet. 1973 12;1(7811):1013-6.	UK	UK	81	1.8
79	Dahiya R, Keller JD, Litofsky NS, Bankey PE, Bonassar LJ, Megerian CA. Temporal bone fractures: otic capsule sparing versus otic capsule violating clinical and radiographic considerations. J Trauma. 1999;47(6):1079-83.	USA	USA	81	4.5
80	Roth M, Lanza DC, Zinreich J, Yousem D, Scanlan KA, Kennedy DW. Advantages and disadvantages of three-dimensional computed tomography intraoperative localization for functional endoscopic sinus surgery. Laryngoscope. 1995;105(12 Pt 1):1279-86.	USA	USA	81	3.7
81	Goldhammer Y, Smith JL. Optic nerve anomalies in basal encephalocele. Arch Ophthalmol. 1975;93(2):115-8.	USA	USA	80	1.9
82	Spetzler RF, Wilson CB. Management of recurrent CSF rhinorrhea of the middle and posterior fossa. J Neurosurg. 1978;49(3):393-7.	USA	USA	80	2.1
83	Wax MK, Ramadan HH, Ortiz O, Wetmore SJ. Contemporary management of cerebrospinal fluid rhinorrhea. Otolaryngol Head Neck Surg. 1997;116(4):442-9.	USA	USA	80	4.0
84	Brennan JW, Rowed DW, Nedzelski JM, Chen JM. Cerebrospinal fluid leak after acoustic neuroma surgery: influence of tumor size and surgical approach on incidence and response to treatment. J Neurosurg. 2001;94(2):217-23.	Canada	Canada	79	4.9
85	McCormack B, Cooper PR, Persky M, Rothstein S. Extracranial repair of cerebrospinal fluid fistulas: technique and results in 37 patients. Neurosurgery. 1990;27(3):412-7.	USA	USA	79	2.9
86	Guerin P, El Fegoun AB, Obeid I, Gille O, Lelong L, Luc S, Bourghli A, Cursolle JC, Pointillart V, Vital JM. Incidental durotomy during spine surgery: incidence, management and complications. A retrospective review. Injury. 2012;43(4):397-401.	France	France	79	15.8
87	Becker SS, Jackler RK, Pitts LH. Cerebrospinal fluid leak after acoustic neuroma surgery: a comparison of the translabyrinthine, middle fossa, and retrosigmoid approaches. Otol Neurotol. 2003;24(1):107-12.	USA	USA	78	5.6
			Canada		
88	Charalampaki P, Ayyad A, Kockro RA, Perneczky A. Surgical complications after endoscopic transsphenoidal pituitary surgery. J Clin Neurosci. 2009;16(6):786-9.	Germany	Germany	77	9.6
89	Hofstetter CP, Singh A, Anand VK, Kacker A, Schwartz TH. The endoscopic, endonasal, transmaxillary transpterygoid approach to the pterygopalatine fossa, infratemporal fossa, petrous apex, and the Meckel cave. J Neurosurg. 2010;113(5):967-74.	USA	USA	77	11.0
90	Schick B, Ibing R, Brors D, Draf W. Long-term study of endonasal duraplasty and review of the literature. Ann Otol Rhinol Laryngol. 2001;110(2):142-7.	Germany	Germany	77	4.8
91	Calcaterra TC. Extracranial surgical repair of cerebrospinal rhinorrhea. Ann Otol Rhinol Laryngol. 1980;89(2 Pt 1):108-16.	USA	‒	76	2.1
92	Selman WR, Spetzler RF, Wilson CB, Grollmus JW. Percutaneous lumboperitoneal shunt: review of 130 cases. Neurosurgery. 1980;6(3):255-7.	USA	USA	75	2.0
93	Leng LZ, Greenfield JP, Souweidane MM, Anand VK, Schwartz TH. Endoscopic, endonasal resection of craniopharyngiomas: analysis of outcome including extent of resection, cerebrospinal fluid leak, return to preoperative productivity, and body mass index. Neurosurgery. 2012;70(1):110-23; discussion 123-4.	USA	USA	75	15.0
94	Ferguson BJ, Wilkins RH, Hudson W, Farmer J Jr. Spontaneous CSF otorrhea from tegmen and posterior fossa defects. Laryngoscope. 1986;96(6):635-44.	USA	USA	75	2.4
95	Gassner HG, Ponikau JU, Sherris DA, Kern EB. CSF rhinorrhea: 95 consecutive surgical cases with long term follow-up at the Mayo Clinic. Am J Rhinol. 1999;13(6):439-47.	USA	USA	75	4.2
96	Mirza S, Thaper A, McClelland L, Jones NS. Sinonasal cerebrospinal fluid leaks: management of 97 patients over 10 years. Laryngoscope. 2005;115(10):1774-7.	UK	UK	75	6.3
97	Luntz M, Balkany T, Hodges AV, Telischi FF. Cochlear implants in children with congenital inner ear malformations. Arch Otolaryngol Head Neck Surg. 1997;123(9):974-7.	USA	USA	74	3.7
98	Papay FA, Maggiano H, Dominquez S, Hassenbusch SJ, Levine HL, Lavertu P. Rigid endoscopic repair of paranasal sinus cerebrospinal fluid fistulas. Laryngoscope. 1989;99(11):1195-201.	USA	USA	74	2.6
99	Cumberworth VL, Sudderick RM, Mackay IS. Major complications of functional endoscopic sinus surgery. Clin Otolaryngol Allied Sci. 1994;19(3):248-53.	UK	UK	74	3.2
100	Drayer BP, Rosenbaum AE. Studies of the third circulation. Amipaque CT cisternography and ventriculography. J Neurosurg. 1978;48(6):946-56.	USA	USA	73	1.9
101	Meurman OH, Irjala K, Suonpää J, Laurent B. A new method for the identification of cerebrospinal fluid leakage. Acta Otolaryngol. 1979 ;87(3–4):366-9.	Finland	Finland	73	1.9

**Table 2:** Numbers of articles in the top 101 list by source Journal.

Journal	N docs	% docs	Impact factor (2017)	JCR category
				Journal category (ranking)
Laryngoscope	20	19.8	2.442	Medicine, Research and Experimental (73/133)
				Otorhinolaryngology (12/41)
Journal of Neurosurgery	17	16.8	4.319	Clinical Neurology (37/197)
				Surgery (14/200)
Neurosurgery	12	6	4.475	Clinical Neurology (36/197)
				Surgery (12/200)
Annals of Otology, Rhinology & Laryngology	6	5.9	1.513	Otorhinolaryngology (22/41)
Otolaryngology-Head and Neck Surgery	6	5.9	2.444	Otorhinolaryngology (11/41)
				Surgery (67/200)
Archives of Otolaryngology-Head & Neck Surgery^a^	4	4.0	3.295	Otorhinolaryngology (12/41)
				Surgery (33/200)
American Journal of Neuroradiology	3	3.0	3.653	Clinical neurology (50/197)
				Neuroimaging (5/14)
				Radiology Nuclear Medicine and Medical Imaging (23/123)
American Journal of Otology^b^	3	3.0	2.182	Clinical Neurology (121/197)
				Otorhinolaryngology (13/41)
American Journal of Rhinology^c^	2	2.0	1.944	Otorhinolaryngology (15/41)
Surgical Neurology d	2	2.0	1.924	Clinical Neurology (139/194)
				Surgery (95/200)
Acta Oto-Laryngologica	1	1.0	1.161	Otorhinolaryngology (29/41)
American Family Physician	1	1.0	1.974	Medicine, General and Internal (58/155)
				Primary Health care (6/19)
American Journal of Roentgenology	1	1.0	3.125	Radiology Nuclear Medicine & Medical Imaging (30/129)
Archives of Ophthalmology^e^	1	1.0	6.669	Ophthalmology (3/69)
British Journal of Surgery	1	1.0	5.433	Surgery (8/200)
Cephalalgia	1	1.0	3.886	Clinical Neurology (43/197)
				Neuroscience (76/261)
Clinical Otolaryngology	1	1.0	2.696	Otorhinolaryngology (7/41)
Expert Review of Proteomics	1	1.0	3.489	Biochemical research Methods (24/79)
Headache	1	1.0	3.091	Clinical neurology (75/197)
Injury-International Journal of the Care of the Injured	1	1.0	2.199	Critical care medicine (25/33)
				Emergency medicine (9/24)
				Orthopedics (30/76)
				Surgery (93/197)
International Forum of Allergy &Rhinology	1	1.0	2.454	Otorhinolaryngology (10/41)
Journal of Clinical Endocrinology & Metabolism	1	1.0	5.789	Endocrinology and Metabolism (20/142)
Journal of Clinical Neuroscience	1	1.0	1.640	Clinical Neurology (156/197)
				Neuroscience (219/261)
Journal of Laryngology and Otology	1	1.0	967	Otorhinolaryngology (36/41)
Journal of Neurology Neurosurgery and psychiatry	1	1.0	7.144	Clinical Neurology (15/197)
				Psychiatry (8/142)
				Surgery (4/200)
Journal of Trauma^f^	1	1.0	3.695	Clinical Care Medicine (10/33)
				Surgery (23/200)
The Lancet	1	1.0	53.254	Medicine, General and Internal (2/155)
Minimally invasive neurosurgery^g^	1	1.0	0. 702	Clinical Neurology (189/197) Surgery (179/200)
New England Journal of Medicine	1	1.0	79.260	Medicine, General and Internal (1/155)
Otolaryngology h	1	1.0	2.444	Otorhinolaryngology (11/41)
				Surgery (67/200)
Otolaryngologic Clinics of North America	1	1.0	1.514	Otorhinolaryngology (21/41)
Otology & neurotology	1	1.0	2.182	Clinical Neurology (121/197)
				Otorhinolaryngology (13/41)
Pituitary	1	1.0	2.730	Endocrinology and Metabolism (86/142)
Radiology	1	1.0	7.469	Radiology, Nuclear Medicine and Medical Imaging (4/121)
World Journal of Surgery	1	1.0	2.766	Surgery (50/200)
World Neurosurgery	1	1.0	1.924	Clinical Neurology (139/197)
				Surgery (95/200)

a Renamed JAMA Otolaryngol Head Neck Surg in 2013.

b Renamed Otology & Neurotology in 2001.

c Renamed American Journal of Rhinology & Allergy in 2009.

d Renamed World Neurosurgery in 2010.

e Renamed JAMA Ophthalmology in 2013.

f Renamed Journal of Trauma and Acute Care Surgery in 2012.

g Renamed Neurochirurgie in 2012.

h Renamed Otolaryngology and Head and Neck Surgery in 1979.

Table 3 lists the JCR categories of the top-cited articles. The leading category was surgery (47.5% of the documents), followed by otorhinolaryngology (46.5%) and clinical neurology (41.6%).

**Table 3:** Distribution of the top 101 cited-papers on cerebrospinal fluid leak group by JCR categories.

JCR category	Documents (n)	Documents (%)
Surgery	48	47.5
Otorhinolaryngology	47	46.5
Clinical neurology	42	41.6
Medicine, research & experimental	20	19.8
Radiology	6	5.9
Nuclear medicine & medical imaging	6	5.9
Neuroimaging	5	5.0
General & internal medicine	3	3.0
Endocrinology & metabolism	2	2.0
Neuroscience	2	2.0
Psychiatry	1	1.0
Biochemical research methods	1	1.0
Primary health care medicine	1	1.0
Ophthalmology	1	1.0
Emergency medicine	1	1.0
Orthopedics	1	1.0

Note: The sum of the percentage was more than 100% because one journal can be included in several categories.

Authors’ country of origin was most commonly the USA (75.2%), followed by the UK (6.9%) and Italy (4%), as shown in Table 4. Most documents were original articles (n = 88), while the other 13 were reviews (n = 12.9). According to the clinical document types assigned in MEDLINE, there were 12 case reports; 4 meta-analyses, and 2 clinical trials.

**Table 4:** Distribution of the top 101 cited papers on Cerebrospinal Fluid Leak group by Country.

Country	N docs	% docs	Documents per 100 million inhabitants
USA	76	75.2	23.23
UK	7	6.9	10.37
Italy	4	4.0	6.61
Canada	3	3.0	8.02
France	2	2.0	3.07
Germany	2	2.0	2.39
India	2	2.0	0.15
South Africa	2	2.0	3.42
Switzerland	2	2.0	23.28
Turkey	2	2.0	2.40
Argentina	1	1.0	2.23
Australia	1	1.0	3.97
Belgium	1	1.0	8.67
China	1	1.0	0.07
Finland	1	1.0	18.08
Spain	1	1.0	2.14
Uganda	1	1.0	2.26

The articles were published between 1954 and 2018, but scientific activity was concentrated between the two decades of 1990–1999 (n = 30) and 2000–2009 (n = 30). Seven of the top papers were published in both 1997 and 1999, while the years 2004 and 2012 each saw the publication of another six top articles (Table 5).

**Table 5:** Distribution of the top 101 cited-papers on Cerebrospinal Fluid Leak group by decade of publication.

Decade	N docs	% docs
1950‒1959	1	1.0
1960‒1969	3	3.0
1970‒1979	14	13.9
1989‒1989	13	12.9
1990‒1999	30	29.7
2000‒2009	30	29.7
2010‒2018	10	9.9

The MeSH terms for the 101 top documents are listed in Table 6. These were led by “Cerebrospinal fluid rhinorrhea” (n = 81). “Cerebrospinal fluid otorrhea” appeared in 33 documents, while “Surgery” was in 62 documents, “Endoscopy” in 35, “Cerebrospinal fluid” in 9, and “Cerebrospinal fluid shunt” in 7 (Table 6).

**Table 6:** Distribution of the 101 top-cited papers on cerebrospinal fluid leak, by medical subject headings (MeSH).

MesH term	N docs	% docs
Cerebrospinal fluid rhinorrhea	81	80.2
Surgery	62	61.4
Aged	50	49.5
Endoscopy	35	34.7
Cerebrospinal fluid otorrhea	33	32.7
Cerebrospinal fluid	9	8.9
Cerebrospinal fluid shunts	7	6.9
Cerebral ventriculography	3	3.0
Anti-bacterial agents	2	2.0
Carotid artery diseases	2	2.0
Brain damage	1	1.0
Acquired immunodeficiency syndrome	1	1.0
Cranial nerve neoplasms	1	1.0

## Discussion

Although several methodologies exist for determining the impact of journals and articles, the number of citations and citation rank lists are still the dominant methods used for identifying influential work in areas including neurosurgery, otolaryngology, ophthalmology, and others.^13^ The analysis of the top-cited articles illustrates how knowledge accumulates over time, therefore this study aimed to determine which articles on CSF leak have had the most influence by ranking the 100 most cited works since 1945. In addition, we analyzed the characteristics of these articles to determine the factors contributing to situating them as the most relevant to other researchers working in the specialty.

The top 101 documents were published in 36 different journals, but half the articles were concentrated in just 5 journals. Otorhinolaryngology and neurosurgery were the dominant specialties, while other disciplines made only nominal contributions to our population of high-impact studies of CSF leak.

Seventy percent of the 101 top-cited articles have been published since 1990, confirming the heavier influence of documents published towards the end of the century. This result is associated with the phenomenon of obsolescence and the concentration of researchers’ interest in more recent studies, as measured through the “half life” of publications, among other citation indicators.^14^

In that regard, scientific evolution in the field of CSF leak has been driven by improvements in diagnostic, imaging, and surgical techniques for its management. One of the most important advances has undoubtedly been the introduction of sinonasal endoscopy for treating the condition.^15^ Endoscopy for fistula closure substantially decreased the morbidity associated with the craniotomy approaches used until the 1940s and increased the closure success rate, which until then had not exceeded 60%.^16^

Since its introduction in the 1970s by Messenklinger and Stammberger,^17^, ^18^, ^19^ endoscopic surgery has progressed tremendously. Beyond its adoption as a treatment for sinonasal pathologies, use of the technique has expanded to other areas, and it is now a major tool for treating skull base pathologies.

Wigang^15^ first discovered endoscopic closure technique in 1981. From then on, the use of this approach spread, and several other authors described their experiences in case series that demonstrated the feasibility and advantages of the method in terms of decreasing morbidity and increasing successful closure.^20^, ^21^, ^22^ These experiences probably explain why a large number of the most cited papers we identified were published over 30 years ago (29%): these studies were pioneering or landmark contributions to the field.

This evolution has been the reason for the high scientific productivity over the years, which corresponds to two different periods: from 1990 to 1999, and from 2000 to 2009. In each of these decades, 30 of the 101 top-cited articles were published.

The first period reflects changes in the management of the fistulas, wherein endoscopic sinus surgery is increasingly favored. Over this decade, articles in high-impact journals described authors’ experiences with innovative techniques.^2^, ^20^, ^23^ This inflection point in the management of the pathology was encapsulated in a meta-analysis published by Hegazy et al. in the year 2000: “Transnasal endoscopic repair of cerebrospinal fluid rhinorrhea: a meta-analysis” in Laryngoscope. In it, the review authors defend and confirm endoscopic surgery as a safe method for CSF fistula closure. This paper had a high impact on the field; the 272 citations it received in the study period make it the second most influential article in our population of documents, with a citation density of 16.^24^

During the second period of highest production (from 2000 to 2009), the topic attracting the most research interest was the expanded endonasal approach to the skull base. Indeed, several articles were published in relation to endoscopic transphenoidal surgery for skull base lesions.^25^, ^26^, ^27^ But an important drawback of this endoscopic approach was the difficulty in reconstructing large dural defects, which often led to complications such as CSF leaks, meningitis or pneumocephalus. For many years, the typical method of closing the dural defects was by means of onlay and inlay grafts, but this technique was associated with very high rates of postoperative leaks.

In this regard, the introduction of the first endonasal pedicle flap, the nasoseptal flap, represented a major impetus to the rapid development and progress of endoscopic skull base surgery. This innovation decreased initial CSF leak rates from 20% to less than 5%, spurring greater expansion of the endoscopic approach.^28^ Thus, the most cited article over the entire study period was by far “A novel reconstructive technique after endoscopic expanded endonasal approaches: vascular pedicle nasoseptal flap”, by Hadad et al., published in 2006 in Laryngoscope.^29^ It also ranked as the article with the highest citation density, at 69.7. The 767 citations it received over the following 12 years illustrates the study’s influence on the evolution of the endoscopic skull base surgery, marking a turning point in the history of the CSF leak surgery, endoscopic endonasal surgery, and endoscopic skull base surgery.

Although the most frequent topic of the top-cited articles was endoscopic surgery, other high-impact articles dealt with other subjects, such as clinical and diagnostic features of CSF leak, neurinoma surgery, or the association between CSF leak and temporal bone fractures. The impact of these articles in the literature peaked from the 1970s to the 1990s; however, after that the important evolution of endoscopic skull base surgery eclipsed the publications focusing on CSF fistulas. In any case, most of the articles presented in this study dealt with surgical aspects of CSF leak, reflecting the wider interest in surgical papers compared to diagnostic or clinical studies.

The USA was the largest contributor to CSF leak research. The vast majority (75.2%) of our high-impact publications come from authors and institutions from that country. This finding is consistent with results reported in other surgical fields such as maxillofacial and plastic surgery.^30^ In addition to the concentration of resources in the USA and its mainstream position in biomedical research, the Matthew effect of accumulated advantage could also be a factor that favors the concentration of citations among journals and authors from this country. Other countries contributing influential papers to the literature include the UK (n = 7), Italy (n = 4), and Canada (n = 3).

The most significant aspect to point out regarding documentary types is the predominance of case studies (35%), with similar values compared to other surgical areas, such as maxillofacial surgery (31%).^31^ Taken alone, clinical case studies do not provide enough evidence for guiding treatment decisions, but when they are collectively considered, appropriately codified and properly integrated into structured information systems, physicians can use the information gleaned as a solid evidence base for comparing cases and checking diagnoses.

The top cited articles were mostly published in otorhinolaryngological and neurosurgical journals, but as in other areas of knowledge, a few generalist journals also stood out for contributing high-impact articles. This is the case of the New England Journal of Medicine, with one document that received 140 citations and had a citation density of 46.7 and an impact factor of 79.260 (2017 JCR).^32^ The Lancet also contributed a document to the list, which garnered 81 citations and showed a citation density of 1,8 and an impact factor of 53.254.^33^

The journal of publication is an important factor determining the potential for an article to be cited.^4^ General medical journals have a wider audience and larger circulation, so they obtain a higher impact factor than smaller specialist journals. Thus, it is difficult to make individual comparisons between journals or to compare the impact of journals from different thematic categories.

There are several limitations to this type of study. First, although citation analysis is one of the most widely used bibliometric parameters, providing a measure of scientific activity, visibility, use, dissemination, and impact, it does not represent a measure of scientific quality.^31^ Second, our search was based on journals with impact factors or under tracking for impact factors. This criterion preferentially favored Western articles, especially those from the USA, the UK, and Canada. Most papers in non-English journals were cited by other papers published in the same language. Therefore, we might have missed a number of highly cited articles related to CSF leak. Third, this study was based on objective citation data, but some landmark CSF leak papers may not have figured among the top-cited papers, as they were cited only until their findings became well known. This phenomenon, termed ‘obliteration by incorporation’, has been observed in other fields. Finally, other factors could also affect the citation rates, such as the journal’s year in review, authors’ self-citations, incomplete citing, and omission bias.^6^ Despite these limitations, citation analysis and impact factor are widely used to rank and evaluate articles and journals. However, these assessment methods should be complemented by others such as peer survey and specialist opinion of citation analysis.

## Conclusion

Our findings offer information related to the dissemination of knowledge in recent decades about the cerebrospinal fluid leaks. Two well-defined periods of maximum scientific activity were driven by surgical innovations. This study also shows that the major specialties contributing to the field of CSF leak were otorhinolaryngology and neurosurgery, which were almost equally represented among the most cited documents.

## Conflicts of interest

The authors declare no conflicts of interest.
